# Supplementary material for: Predicting Daily Dry Matter Intake Using Feed Intake of First Two Hours after Feeding in Mid and Late Lactation Dairy Cows with Fed Ration Three Times Per Day
Source: Animals (Basel). 2021 Jan 6;11(1):104. doi: 10.3390/ani11010104 (PMC7825592; doi:10.3390/ani11010104)
Supplement: Supplementary file 1 [file animals-11-00104-s001.pdf]

Supplementary Table S1. Ingredient composition and nutrient of the total mixed rations.

Ingredient, % DM	DDS[2]	VDS[3]
Alfalfa hay	16.1	6.0
Oat hay	7.55	6.09
Corn silage	18.8	9.80
Brewer grains	3.47	0
Beet pulp	4.67	3.54
Cottonseed mean, whole	5.49	3.96
Steam-flaked corn	5.57	11.0
Total mixed fermentation ration	0	28.0
Ground corn grain	17.0	13.9
Soybean meal	9.62	7.92
Expanded soybean	2.74	2.31
Fat meal	1.09	1.01
Distillers dried grains with soluble	4.62	3.80
$CaHPO_4$	0.11	0.10
NaCl	0.21	0.17
Limestone	0.36	0.29
$NaHCO_3$	0.34	0.27
MgO	0.13	0.11
Premix[1]	2.12	1.74
Nutrient levels		
Dry matter	51.8	50.4
Crude protein	16.0	15.9
Neutral detergent fiber	32.9	34.7
Acid detergent fiber	19.0	21.1
Organic matter	95.5	96.2
NEL, Mcal/kg DM	1.70	1.70

[1] Premix, formulated to provide (per kg of DM): vitamin A ≥ 600 kIU, vitamin D3 ≥ 150 kIU, vitamin E ≥ 2,000 IU, nicotinic acid ≥500 mg, Cu ≥ 1500 mg, Fe ≥ 1,500 mg, Mn ≥ 1,500 mg, Zn ≥ 7,000 mg, I ≥ 90 mg, Se ≥ 50 mg, Co ≥ 20 mg.

[2] DDS = development dataset

[3]VDS = validation dataset

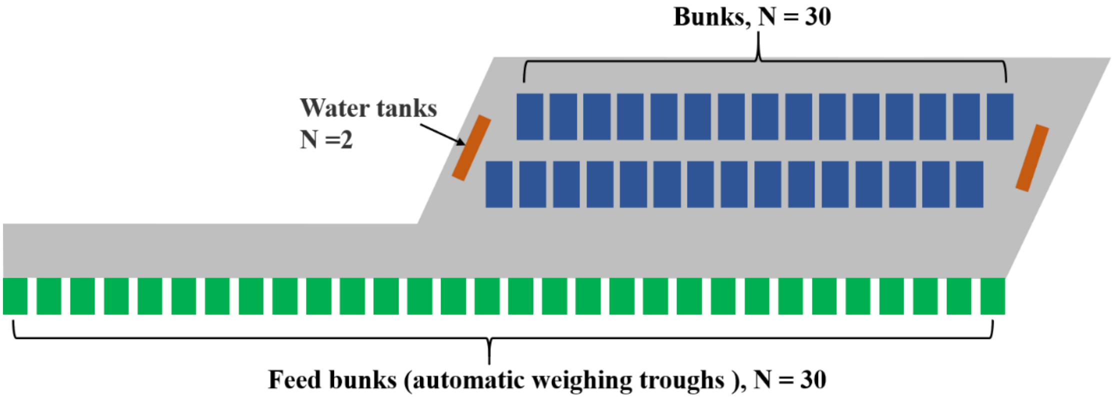

Supplementary Figure S1. The ichnography of feed bunk and barn.

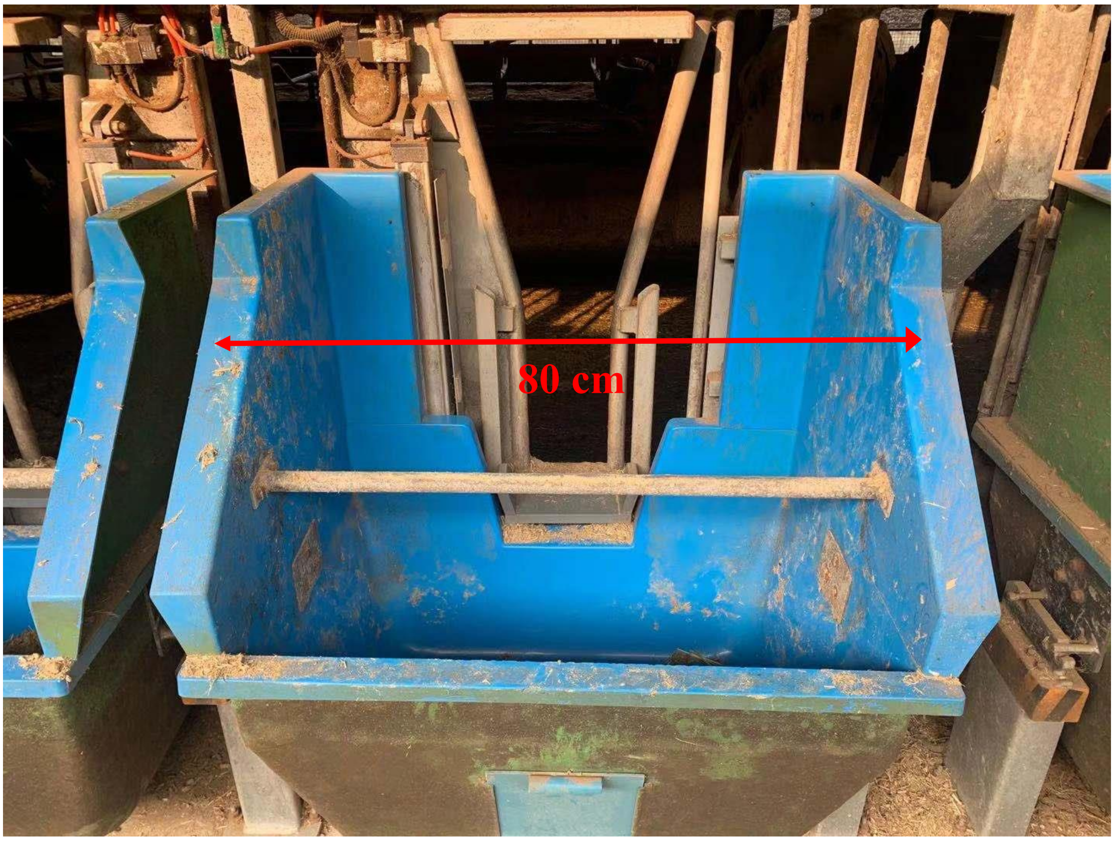

Supplementary Figure S2. The feed bunk (automatic weighing trough), width is 80 cm.
